# T-cell reprogramming through targeted CD4-coreceptor and T-cell receptor expression on maturing thymocytes by latent *Circoviridae* family member porcine circovirus type 2 cell infections in the thymus

**DOI:** 10.1038/emi.2015.15

**Published:** 2015-03-11

**Authors:** Stefanie Klausmann, Titus Sydler, Artur Summerfield, Fraser I Lewis, Roseline Weilenmann, Xaver Sidler, Enrico Brugnera

**Affiliations:** 1 Institute of Veterinary Pathology, Vetsuisse Faculty, University of Zurich, CH-8057 Zurich, Switzerland; 2 Department of Farm Animals, Division of Swine Medicine, Vetsuisse Faculty, University of Zurich, CH-8057-Zurich, Switzerland; 3 Institute of Virology and Immunology (IVI), 3147 Mittelhäusern, Switzerland; 4 Section of Epidemiology, Vetsuisse Faculty, University of Zurich, CH-8057 Zurich, Switzerland

**Keywords:** adaptive immune response failure, CD4^+^ thymocyte maturation diversion, *Circoviridae* porcine circovirus type 2 pathogenicity, dendritic cell feedback, *in vivo* anergy, polyclonal negative selection, T-helper cell hypo-responsiveness, thymic kinetic signaling model

## Abstract

Although porcine circovirus type 2 (PCV2)-associated diseases have been evaluated for known immune evasion strategies, the pathogenicity of these viruses remained concealed for decades. Surprisingly, the same viruses that cause panzootics in livestock are widespread in young, unaffected animals. Recently, evidence has emerged that circovirus-like viruses are also linked to complex diseases in humans, including children. We detected PCV2 genome-carrying cells in fetal pig thymi. To elucidate virus pathogenicity, we developed a new pig infection model by *in vivo* transfection of recombinant PCV2 and the immunosuppressant cofactor cyclosporine A. Using flow cytometry, immunofluorescence and fluorescence *in situ* hybridization, we found evidence that PCV2 dictates positive and negative selection of maturing T cells in the thymus. We show for the first time that PCV2-infected cells reside at the corticomedullary junction of the thymus. In diseased animals, we found polyclonal deletion of single positive cells (SPs) that may result from a loss of major histocompatibility complex class-II expression at the corticomedullary junction. The percentage of PCV2 antigen-presenting cells correlated with the degree of viremia and, in turn, the severity of the defect in thymocyte maturation. Moreover, the reversed T-cell receptor/CD4-coreceptor expression dichotomy on thymocytes at the CD4^+^CD8^interm^ and CD4SP cell stage is viremia-dependent, resulting in a specific hypo-responsiveness of T-helper cells. We compare our results with the only other better-studied member of Circoviridae, chicken anemia virus. Our data show that PCV2 infection leads to thymocyte selection dysregulation, adding a valuable dimension to our understanding of virus pathogenicity.

## INTRODUCTION

Life is dependent on a fine-tuned immune system that balances self-tolerance and recognition of foreign antigens. T-cell maturation in the thymus is central to these distinctions. Any disturbance of this system during differentiation and maturation renders affected individuals susceptible to infection, autoimmunity, allergies, tumors and even aging. In the thymus, alphabeta- and a minority of gammadelta-T cells express the T-cell receptor (TCR) and the associated CD3 chains, including CD3ε. Alphabeta-T cells additionally express the CD4 and/or CD8 coreceptors that, together with the TCR, form the signaling module central to their maturation and peripheral T-cell function.^1^ CD8-coreceptor-expressing T cells interact with major histocompatibility complex (MHC) class-I (MHC-I) presented ligands, and CD4-coreceptor expressing T cells interact with MHC class-II (MHC-II) presented ligands. During thymocyte maturation, CD4 and CD8 coreceptor double-positive (DP) T cells mature by migrating from the thymic cortex to the corticomedullary junction, leading to coreceptor single-positive (SP) T cells in the medulla. Thymocytes with the appropriate self-reactive signaling-module avidity survive by positive selection, and thymocytes with strong avidity are generally eliminated by negative selection.^2^ Thymocytes that receive insufficient signals undergo death by neglect.^2^ The signal strength is mostly dependent on the signaling module interaction with self-ligand-loaded MHC presented by thymic epithelial cells (TECs) and dendritic cells that migrate into the thymus.^3^ Notably, persistent TCR and coreceptor signals favor CD4SPs and the cessation of coreceptor signaling results in CD8SPs.^4,5^ Both lineages mature through the CD4^+^CD8^interm^ or even CD4SP stage, as described in the kinetic signaling model.^4,5^ These naive T cells ^6^ with thymic predetermined T-cell specificity are tested again peripherally for self-reactivity. Self-ligand loaded MHCs causing persistent or strong signals through the signaling module provoke *in vivo* T-cell anergy, also known as adaptive tolerance, in the periphery.^7^

*Circoviridae*^8,9^ and the closely related *Anelloviridae* (torque teno virus),^10^ are found abundantly in animals ^9,11,12^ and humans.^9,12,13,14,15^ Coinfections with both *Circoviridae* and *Anelloviridae* family members are common ^16,17^ and reciprocally enhance their pathogenicity.^18^ In the last two years, it has become apparent that *Circoviridae* is also associated with human diseases, including those in children.^12,15,19,20^ Low virus concentrations are common in healthy individuals, and higher virus concentrations are disease-associated. In pig and poultry livestock, *Circoviridae* was found to be responsible for panzootics.^21,22^ The typical family member, porcine circovirus type 2 (PCV2), seems to be essential and yet not sufficient by itself to induce disease.^23^ An icosahedral capsid protects a small circular and single-stranded DNA genome of the infectious particle.^21^ The viral double stranded DNA (dsDNA) is indicative of viral replication and possible capsid production.^24,25^ The NCBI databases contain several hundred PCV2 sequences belonging to four genotype groups,^26^ of which the PCV2a and PCV2b genotypes dominate.^27,28^ A robust, reliable pig infection model ^29^ is a major challenge, as immune modulatory cofactors are needed to induce disease.^30,31^ In fact, both genotypes seem to be required for virus replication and may also enhance pathogenicity.^32,33^ PCV2 association with several complex diseases, including postweaning multisystemic wasting syndrome (PMWS), now renamed PCV2-systemic disease (PCV2-SD),^34^ has become infamous in pig-producing countries.^35^ In healthy pigs, <10^6^ PCV2 genomes/mL blood are common, and more than 10^7^ genomes/mL blood^35^ or moderate to high levels of PCV2 antigen in the secondary lymph organs are associated with disease.^35,36^ In pigs, enlarged lymph nodes,^37^ lymphopenia (T- and B-lymphocyte depletion),^38^ wasting and diarrhea are obvious signs of disease.^31^ Although accelerated thymus atrophy^39^ was noted in PCV2-SD, the main focus to date has remained secondary lymphoid organ infections.

The aim of our study was to understand the fundamentals of the pathogenicity of the *Circoviridae* family member PCV2. We developed a new PCV2 pig infection model that took advantage of the immune suppressant cofactor cyclosporine A (CsA) to follow PCV2 productive infection through PCV2-SD manifestation. More importantly, we demonstrate that PCV2 strongly impacts T-cell selection processes in the thymus. These data unexpectedly reveal for the first time that PCV2 takes advantage of thymic developmental processes that ensure the balance between self-tolerance and immune defense against pathogens.

## MATERIALS AND METHODS

### Ethics statement

The animal experiments and protocols were approved by the Animal Welfare Committee of the Canton Bern (authorization NO 98/09 and license BE26/11) and by the Animal Welfare Committee of the Canton Zurich (authorization NO 227/2010), respectively. Handling of and experiments with pigs were carried out in accordance with EU standards and the Swiss Animal Welfare law (Tierschutzgesetz SR455).

### Pig experiments and treatment

We present data primarily from three independent pig infection experiments at the Institute of Virology and Immunology (IVI, Mittelhäusern, Switzerland) and at the Institute of Veterinary Pathology (Zurich, Switzerland).

To establish the *in vivo* effectiveness of CsA (Sandimmun Neoral^®^ Novartis, Basel, Switzerland), we prepared the pigs' peripheral blood lymphocytes (PBLs) and cultured them *in vitro* with or without proliferative agents. PBLs from CsA-treated pigs were hyper-responsive when *in vitro* stimulated with concanavalin A (ConA) (Supplementary Figure S1).

Six 4-week old weaners, purchased from a Swiss breeding farm at 3 weeks of age, were randomly grouped. Three weaners received 30 mg/kg/day CsA and were separately *in vivo* transfected with 4 μg, 8 μg or 20 μg recombinant PCV2 DNA cocktail (PCV2a/PCV2b at a 1:2 ratio). Three weaners were mock transfected, and two of these three also received 30 mg/kg/day CsA until necropsy 30 days post-transfection (p.t.). The PCV2 DNA cocktail-injected and immunosuppressed weaners were kept isolated from those treated with CsA only or the control weaner. We measured the centrifollicular PCV2-antigen staining in the tonsil to determine whether recombinant PCV2 cocktail and CsA treatment induced persistent infections.

In another pig infection experiment, 12 4-week-old weaners (in house-bred, Swiss Large White) were randomly arranged into 4 groups and kept separately in isolated rooms at the IVI. Eight pigs were *in vivo* transfected with 20 μg DNA cocktail of both PCV2 genotype group members, PCV2a and PCV2b, by direct injection into the weaners' neck region. Four of these eight pigs also received 30 mg/kg/day CsA. The remaining four were mock transfected, with two receiving CsA only and two weaners serving as untreated controls.

To repeat and extend the previous data, we conducted another pig infection experiment. Fourteen 4-week-old weaners, purchased from a Swiss breeding farm at age 3 weeks, were randomly divided into four groups. Again, eight weaners were *in vivo* transfected with the DNA cocktail encoding both PCV2 genotype group members. Four of the eight PCV2-transfected weaners also received CsA. The remaining six were mock transfected, with three receiving CsA only and three weaners serving as untreated controls.

All pigs that were CsA treated received 30 mg/kg CsA daily orally until necropsy. The purchased weaners were Swiss Large White from a well-managed Swiss breeding farm^36^ with no clinical signs of disease (no porcine circovirus disease history) and an absence of major respiratory and intestinal pathogens affecting piglets.^36^ Our antibodies in the flow cytometry analysis could not detect a variation in possible CD4-coreceptor haplotypes^40^ in pigs. Temperature and clinical signs were recorded daily in a scoring system (appetite, liveliness, thinness, skin color, diarrhea and breathing),^41^ and body weight was also documented every second to third day. The weaners were slaughtered by electrical stunning or euthanized according to standard protocols and immediately necropsied.

### Pig organs and blood handling

Organs and blood samples were immediately collected after euthanization and further processed. PBLs or peripheral blood mononuclear cells (PBMCs) were isolated from the blood and used further for either flow cytometry analysis or *in vitro* assays. Tissue samples from the thymus, spleen, inguinal lymph node, mesenteric lymph node, ileum, heart, lung, kidney, liver, small intestine and colon were taken at necropsy and fixed in formalin or hepes glutamic acid buffer-mediated organic solvent protection effect (HOPE, Polysciences Inc. DCS, Hamburg, Germany) fixative.

### Construction of recombinant PCV2 genotype group members

PCV2 DNA was isolated from different paraffin-embedded pig tissue blocks that were previously defined according to their PCV2 genotype.^36^ PCV2-specific DNA fragments were amplified by polymerase chain reaction (PCR) using the proofreading polymerase Vent (New England BioLabs, Allschwil, Basel-Country, Switzerland) and ligated into pCR2.1-TOPO vector (Invitrogen, Basel, Switzerland). We constructed 24 whole virus genomes and obtained two new PCV2a genotype group members (JX512853 and JX512854) and six new PCV2b genotype group members (JX512855, JX512856, JX512857, JX512858, JX512859 and JX512860) that were deposited into the NCBI database.

### *In vivo* transfection using JetPei-carrier

Equal concentrations of three PCV2a, JX512853, JX512854 and Stoon10-10 (each 2.22 μg), and the six PCV2b genotype group members (2.22 μg each) were mixed. The resulting PCV2 DNA cocktail was split equally for separate incubations with the DNA carriers JetPei or JetPei-Man (Polyplus transfection, Illkirch, France) in glucose. After incubation, the PCV2 DNA carrier cocktail was mixed for injection on both sides of the weaner's neck region near the dorsal superficial cervical lymph node. The CysA and control groups were mock *in vivo* transfected with carrier.

### Antibodies, proliferation dye and detection of apoptotic cells and flow cytometry analysis

Two 3-color stainings were carried out at the IVI for detection of T and B cells. For T cells, we used mouse anti-porcine CD3ε (clone PTT3, a generous gift from Dr. Armin Saalmüller, Institute of Immunology, University of Veterinary Medicine, Vienna) followed by goat-anti mouse IgG1-fluorescein isothiocyanate (FITC, Southern Biotech, Allschwil, Basel-Country, Switzerland), mouse anti-porcine CD4α (clone 74-12-4, a generous gift from Dr A. Saalmüller, Vienna) bound with goat-anti mouse IgG2b-R-phycoerythrin (RPE, Southern Biotech, Switzerland) and mouse anti-porcine CD8α (clone 76-2-11, a generous gift from Dr. Armin Saalmüller, Vienna) followed by goat-anti mouse IgG2a-biotin (Southern Biotech) plus streptavidin-SpectralRed (Southern Biotech). For B-cell detection, we used mouse anti-porcine CD52 (clone HB 141, a generous gift from Dr A. Saalmüller, Vienna) bound with goat-anti mouse IgM-FITC, mouse anti-porcine CD172a (clone 74-22-15A, a generous gift from Dr A. Saalmüller, Vienna) bound with goat-anti mouse IgG2b-RPE (Southern Biotech) and mouse anti-porcine CD14 (clone CAM36A, a generous gift from Dr A. Saalmüller, Vienna) bound with goat-anti mouse IgG1-biotin (Southern Biotech) plus streptavidin-SpectralRed (Southern Biotech, Switzerland). Experiments at the Institute of Veterinary Pathology (Zurich, Switzerland) were performed to detect T cells with mouse anti-pig CD3ε-FITC (clone BB23-8E68C8, BD, Allschwil, Basel-Country, Switzerland), mouse anti-porcine CD4α-peridinin chlorophyll protein (PerCP-Cy 5.5, clone 74-12-4; BD), mouse anti-pig CD8α-PE (clone 76-2.11; BD, Switzerland) and mouse anti-porcine CD25 (clone MCA1736, AbD Serotec, Puchheim, Bavaria, Germany) visualized with allophycocyanin (APC)-coupled rat anti-mouse IgG1 (clone A85-1; BD). The same 4-color antibody combination and Celltrace violet stains (Invitrogen Life Technologies, Wohlen, Aargau, Switzerland) were used to visualize lymphocyte proliferation. To detect apoptotic cells, we used propidium iodide (PI, Sigma- Aldrich, Buchs, St. Gallen, Switzerland) and annexin V-FITC (BD) in cell suspension.

For acquisition of data, a FACSCalibur with CellQuest software (BD) at the IVI and a FACS-Canto II with FACSDiva software (BD) at the Flow Cytometry Facility (University of Zurich) were used. *In vitro* assays were performed and analyzed in Zurich. Pig B cells were analyzed at the IVI. The hypo-responsiveness of T-helper cells (Ths) as well as TCR/CD4-coreceptor dichotomy were detected in both locations. Data points were normalized to the controls and presented.

Forward light scatter, side light scatter and propidium iodide staining were used to exclude dead cells. The analysis was performed with the help of FlowJo vX (Treestar, Ashland, Oregon, United States) software.

### Thymocyte preparation, culture and cell proliferation

Thymocytes were separated from fresh pig thymi. All pig cells were counted before and after the experiments using the Trypan Blue exclusion assay.

Pig lymphocytes were cultured at 39 °C in 7.5% CO_2_ humidified air atmosphere.

For the proliferation assays, isolated PBMCs were stained with Celltrace violet stain (Invitrogen Life Technologies) and incubated with ConA or phorbol 12-myristate 13-acetate (PMA, Sigma- Aldrich, Buchs, St. Gallen, Switzerland) and ionomycin (IO; Sigma-Aldrich, Switzerland) or cultured without proliferative agents for 68 h.

### Anti-PCV2 IgG and IgM enzyme-linked immuno assays

Enzyme-linked immuno assays (SERELISA^®^ PCV2b AB Mono Blocking, SYNBIOTICS EUROPE SAS 2, Lyon, Rhone, France) and INGEZIM Circovirus IgG/IgM (Ingenasa, Madrid, Spain) were used as described.^42^ Nine or 10 consecutive blood samples were taken from each pig over 31 or 52 days to determine the antibody content and viremia.

### SYBR Green-based real-time PCR

**Figure 1:**
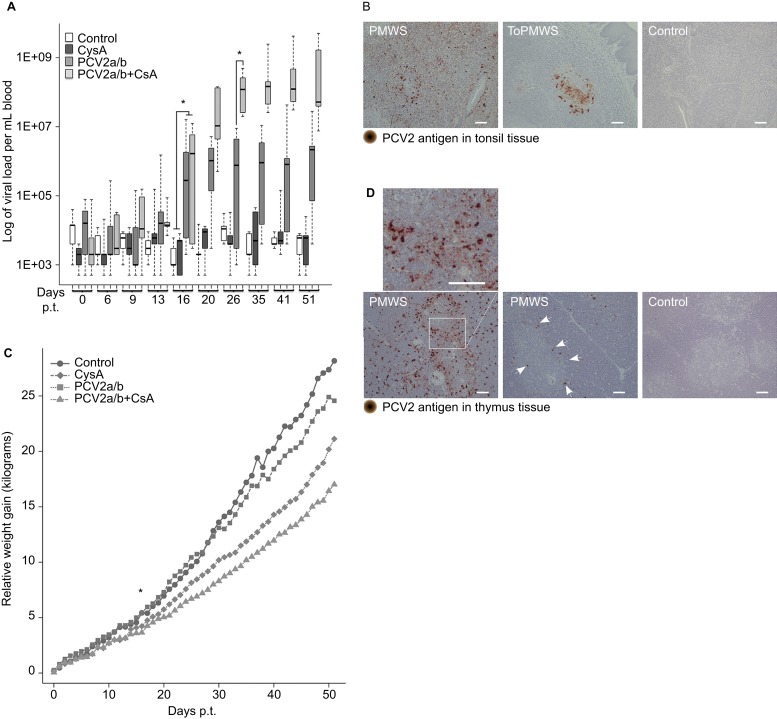
PCV2 *in vivo* transfection and CsA synergistically caused PCV2-SD. (**A**) PCV2 viremia in conventional pigs at various days p.t. PCV2 genome real-time PCR was determined from whole blood. PCV2a/b (*n*=9) or PCV2a/b+CsA (*n*=5) compared with CysA (*n*=5) or control (*n*=5) pig viremia were significantly different from day 16 p.t. (pigs per group (*n*); *P<0.003, Kruskal–Wallis rank sum test). PCV2a/b and PCV2a/b+CsA pig viremia became different at day 26 p.t. (*P<0.001, Kruskal-Wallis rank sum test). (**B**) Paraffin-embedded tonsil tissue with PCV2-antigen staining. Paraffin-embedded thymus tissue sections counterstained with hematoxylin (blue nucleus) and 100 μm white bars. (**C**) PCV2 infection hinders daily weight gain of pigs. The average weight gain of PCV2-transfected pigs was significantly different from CysA or control pigs by day 17 p.t. (*P<0.04, Kruskal–Wallis rank sum test). (**D**) PMWS pigs with different thymic PCV2-antigen concentrations. White arrows indicate PCV2-antigen at the corticomedullary junction. Control PCV2-antigen concentrations are representative of the other pig groups (ToPMWS, PCV2a/b and CysA pig group). Paraffin-embedded thymus tissue sections counterstained with hematoxylin (blue nucleus) and 100 μm white bars.

We used a real-time PCR method based on oligonucleotides and plasmid standards^36^ to determine the blood PCV2 DNA template concentrations. Because we used a logarithmic scale and the original numbers were based on microliters but were then calculated in milliliters, we replaced the value of 0 virus genomes with 499 in Figure 1A.

### Immunohistochemistry (IHC)

IHC was carried out as previously described on tissue sections labeled with anti-PCV2 antibody.^33^ HOPE-fixed tissue sections were directly stained with anti-CD8β (Clone 295/33-25; BD) antibody. We used mild alkaline treatment for mouse anti-equine monoclonal MHC-II (LS-C124117-LSBio, Lab Force AG, Nunningen, Solothurn, Switzerland) or HOPE-fixed tissue for mouse anti-porcine MHC-II (MSA3, Kingfisher, Hamburg, Germany) staining.

### Fluorescence *in situ* hybridization (FISH) combined with immunofluorescence (IF)

The signal specificity of oligonucleotide-mediated FISH was previously established and described in detail.^33^ We used the oligonucleotides P2O-O1r (5′-GCA TGT TGC TGC TGA GGT GCT GCC G-3′) labeled at the 5′ and 3′ ends with Dyomics 630 and/or the reverse complement of P2O-O1f labeled at the 5′ and 3′ ends with ATTO 565 (Microsynth AG, Switzerland) for FISH experiments. IF combined with FISH tissue sections were overlaid first with their respective antibodies (see IHC) and followed with biotinylated polyclonal goat anti-mouse immunoglobulins (Dako, Baar, Zug, Switzerland) in 0.5% pig sera to avoid any nonspecific binding of the antibodies. The antibody complex was fixed to the tissue with the help of 3.7 % formaldehyde, (Merck, Zug, Switzerland), 17% glacial acetic acid (Merck) and 50 % ethanol (Merck). After extensive washing, the tissue sections were FISH stained, and finally, the antigens were visualized with Alexa Flour 568 streptavidin (Invitrogen).

### Statistical analyses

The R statistical software environment (R Core Team 2013) was used for all data modeling. Because of the potential for correlated observations (multiple observations from the animal), generalized linear mixed models were utilized where appropriate using the lme4 and nlme extension packages for R. For analyses with only one observation per animal, the non-parametric Kruskal–Wallis rank sum test was used to identify treatment (group) effects. A *P* value of ≤0.05 was accepted as statistically significant.

## RESULTS

### The new pig infection model: productive PCV2 infection, together with the cofactor CsA, leads to PCV2-SD development

We used recombinant PCV2 group members for *in vivo* transfection to avoid virus passage artifacts. PCV2 infection persistency was tested in the presence of the cofactor CsA. *In vivo* PCV2-transfected and CsA-treated pigs carried PCV2-antigen in the tonsil for 30 days p.t. These pigs were viremic and PCV2-specific IgM antibody positive, and one of three had low concentrations of PCV2-specific IgG (Table 1) comparable to subclinically infected pigs on farms.^42^

Pigs from all trials were graded based on the viral antigen concentrations in the secondary lymphoid organs and PCV2 viremia. In five male pigs in the PCV2a/b+CsA group, low to very high PCV2-antigen concentrations were found in the tonsils and other secondary lymphoid organs (Figure 1B and Table 2) 51 days p.t. Two of these pigs, which also belonged to the PMWS group, with approximately 10^9^ viruses/mL blood showed clinical PCV2-SD signs of diarrhea (PCV2-antigen in the intestinal lamina propria and Peyer's patches, Table 2), reduced body weight, thinner appearance and contained moderately high to very high PCV2-antigen in the secondary lymphoid organs (Figure 1B and Table 2) 51 days p.t. The other three pigs, called the ToPMWS group, with 10^7^–10^8^ viruses/mL blood, contained low to moderately high PCV2-antigen in the tonsils and secondary lymphoid organs (Figure 1B and Table 2). The PCV2a/b group consisted of nine pigs that, over the 52-day period, presented over 10^6^ viruses/mL blood at least once or low or no antigen in the secondary lymphoid organs (Table 2). Six of these nine pigs were only transfected with virus cocktail and did not receive CsA. One male and two PCV2-transfected females treated with CsA were classified as belonging to the PCV2a/b group according to the low PCV2-antigen content of the secondary lymphoid organs.

The PCV2a/b+CsA pigs' viremia (Figure 1A) was similar to disease-affected pigs on farms.^31^ The first virus concentration over the background was measured in a PCV2a/b pig only 13 days p.t. (Figure 1A). Moreover, the viremia in PCV2-transfected pigs was significantly higher from day 16 p.t. when compared to CysA or control group pigs that were not additionally PCV2-transfected (Figure 1A, *P*<0.003). The CsA immunosuppression synergy with PCV2 infection was measured from day 26 p.t., as the viremia in PCV2a/b and PCV2a/b+CsA pigs became significantly different (Figure 1A, *P*<0.001). The average viremia in the presence of CsA immunosuppression increased 70 times over the viremia in PCV2-only transfected pigs.

PCV2 *in vivo* transfections generally lowered the average weight gain (Figure 1C), similar to field studies.^42^ The PCV2a/b+CysA (*n*=5) and PCV2a/b (*n*=9) pigs gained less weight on average, at 14.8±3.1 kg and 18.8±4.8 kg, respectively, compared to 28.2±2.9 kg in the control pigs (*n*=3) and 24.6±4.8 kg in the CysA pigs (*n*=3) over 52 days. The PCV2-transfected pig average weight gain was significantly different from day 17 p.t. (*P*<0.04).

Initial recognition of PCV2 infection by the immune response seemed normal judged by the appearance of PCV2-specific IgM antibodies in all 13 males and 3 of 6 female pigs upon transfection (Table 1). We found that female pigs were generally less susceptible to PCV2 infections (Table 1). Two PCV2 *in vivo*-transfected females never responded to PCV2 transfection and were excluded from the pig groups (Table 1). Additionally, two female pigs that were *in vivo* transfected with PCV2 and treated with CsA responded similarly to PCV2-only transfected males. Only one female from nineteen pigs developed any notable concentration of PCV2-specific IgGs over the background after PCV2 transfection and CsA treatment (Table 1). Host genetics seem to play a role in disease development,^31,43^ which is not unique to PCV2-associated diseases, as this has also been observed for other viral diseases.^44^

Although IgM antibodies persisted in 6 of the 12 male pigs until the day of slaughter (51 p.t.), viremia was not significantly reduced (Figure 1A and Table 1). Furthermore, B-cell (defined as CD52^−^, CD172a^−^ and CD14^−^) cellularity in eight investigated PCV2-transfected males (ToPMWS (*n*=3), PCV2a/b (*n*=5)) was not different from the CysA (*n*=2) or control (*n*=2) groups when compared in 10 consecutive blood samples over 52 days. The observed suboptimal B-cell response parallels observations that subclinically infected pigs cannot be diagnosed by PCV2-specific IgGs^45^ and that PCV2-SD affected pigs often do not have PCV2-specific IgG antibody responses in field studies.^46^

Notably, only thymi from PMWS pigs contained PCV2 capsid protein, mostly at the corticomedullary junction of the thymus (Figure 1D). The pig from the PMWS group that had more viral antigen staining in the secondary lymphoid organs (Table 2) and an earlier onset of diarrhea (at day 49 of 52) contained remarkably more PCV2-antigen in the thymus (Figure 1D) than the pig that developed diarrhea at day 51. Dominant viral antigen staining was found on single cells (Figure 1D). The staining followed the shape of these cells relatively well. These cells appeared polygonal with possible dendritic extensions (Figure 1D). The cell type best resembled APCs, which are seen in the mice thymus as new immigrating dendritic cells presenting self-ligand.^3^ In contrast, the pigs (*n*=30) that were not diagnosed with PCV2-SD did not contain any detectable viral antigen staining in the thymus (Figure 1D and Table 2).

### Specific polyclonal deletion of SPs is associated with thymic PCV2-antigen presentation by APCs, implicating negative selection in the thymus

**Figure 2:**
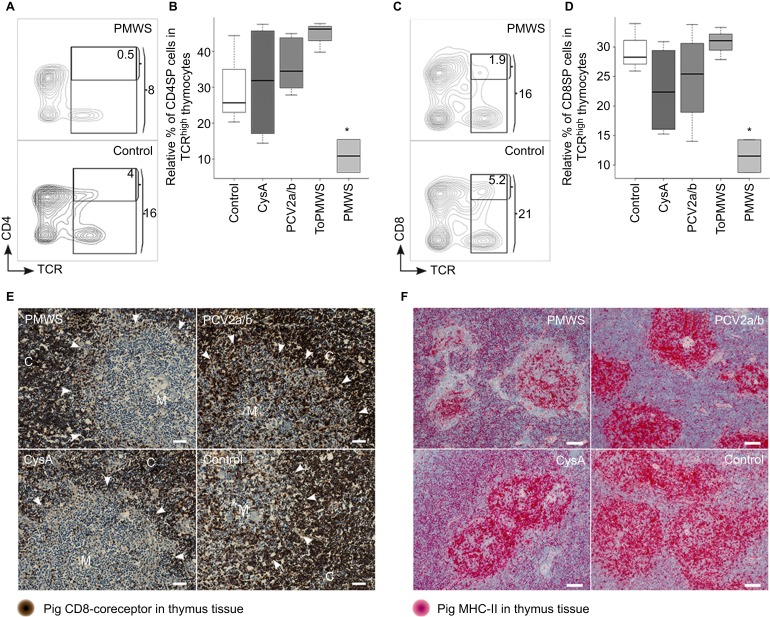
CD4^+^TCR^high^ thymocytes and CD8^+^ TCR^high^ thymocytes are polyclonally negatively selected in thymus of PMWS pigs. (**A**–**D**) Specific SP cell depletion in the thymi of diseased pigs. (**A**) Contour plot of CD4 coreceptor versus TCR-expressing thymocytes with the indicated gates (percentage numbers of total thymocytes). (**B**) Comparison of CD4SPs to total TCR (CD3ε) expressing thymocytes. PMWS (*n*=2) contained significantly fewer CD4SPs (Control (*n*=3), CysA (*n*=4), PCV2a/b (*n*=9), ToPMWS (*n*=3); pigs per group (*n*); *P<0.03, Kruskal–Wallis rank sum test). (**C**) Contour plot of CD8 coreceptor versus TCR expressing thymocytes with the indicated gates (percentage numbers of total thymocytes). (**D**) Comparison of CD8SPs to total TCR (CD3ε) expressing thymocytes. PMWS (*n*=2) contained significantly fewer CD8SPs (Control (*n*=3), CysA (*n*=4), PCV2a/b (*n*=9), ToPMWS (*n*=3); pigs per group (*n*); *P<0.03, Kruskal–Wallis rank sum test). (**E**) Thymic medullar CD8^+^ thymocyte depletion in PMWS pigs. Paraffin-embedded thymus tissues counterstained with hematoxylin (blue nucleus) and 50 μm white bars. White arrows indicate the corticomedullary junction, the white M indicates the thymus medulla, and the white C indicates the thymus cortex. (**F**) Ring-shaped MHC-II cell depletion around the corticomedullary junction in PMWS thymi. Paraffin-embedded thymus tissue sections counterstained with hematoxylin (blue nucleus) and 100 μm white bars.

We investigated whether the new occurrence of PCV2-loaded APCs may have effects on the thymocyte populations of the PMWS thymi. We found a specific reduction in CD4^+^ TCR^high^ cells, from 4% to 0.5% of the total thymocytes (Figure 2A). Additionally, CD4SPs were also significantly reduced within the TCR^high^ expresser thymocytes when compared to the CD4SP occurrence within the TCR^high^ thymocytes of the remaining 4 groups (Figure 2B). In parallel, the CD8^+^ TCR^high^ cells in the PMWS pigs were also reduced, from 5.2% to 1.9% of the total thymocytes (Figure 2C). Again, within the TCR^high^ expressers, we observed a significant reduction in the CD8SPs in the PMWS thymi (Figure 2D), similar to the reduction in CD4SPs. We did not find any significant depletion of the DPs (data not shown).

These data were also supported by the specific absence of CD8^+^ thymocytes in the medulla of diseased pigs by immunohistochemistry (Figure 2E). Comparing the CD8-coreceptor staining intensity of the thymus tissue from PMWS (*n*=2) with control (*n*=3) or PCV2a/b (*n*=6) pigs, we noticed its absence in the medulla of the thymus. Although the thymic medullae of the PMWS pigs were homogeneously depleted, the thymic medullae in CysA pigs (*n*=3) was mosaically depleted for the CD8 coreceptor. Approximately 10%–40% of the medullae in the CysA pigs' thymi were depleted, and the rest of the medullae appeared similar to the control medullae (Figure 2E). These data together indicate specific polyclonal depletion of the maturing SPs, which resembles thymic negative selection.

As clonal deletion in negative selection is driven by APCs presenting self-ligand, we analyzed the presence of APCs immunohistochemically using MHC-II expression. Notably, TECs, APCs and a subpopulation of maturing DPs expressing mainly MHC-II were present in the porcine thymus.^47^ Using two different antibodies against porcine MHC-II, we observed that the thymic cortex was well structured with a fine scaffold of cortical TECs visible particularly in the CsA-treated pigs (*n*=18) (Figure 2F). Thymic medulla (*n*=32) staining appeared stronger and more granular (Figure 2F). Interestingly, we observed a deficiency of MHC-II expressing cells around the thymic corticomedullary junction that appeared to be ring-shaped (Figure 2F) in only the PMWS pigs (*n*=2). This deficiency seemed thymic viral antigen concentration-dependent compared to the other pig thymi (*n*=30), with no detectable antigen (Figures 1D and 2F). Moreover, the pig with more thymic viral antigen (Figure 1D) showed a more pronounced ring structure (data not shown). The absence of MHC-II-expressing cells indicates a possible link between SP depletion and PCV2-antigen expression and presentation in the thymus.

**Figure 3:**
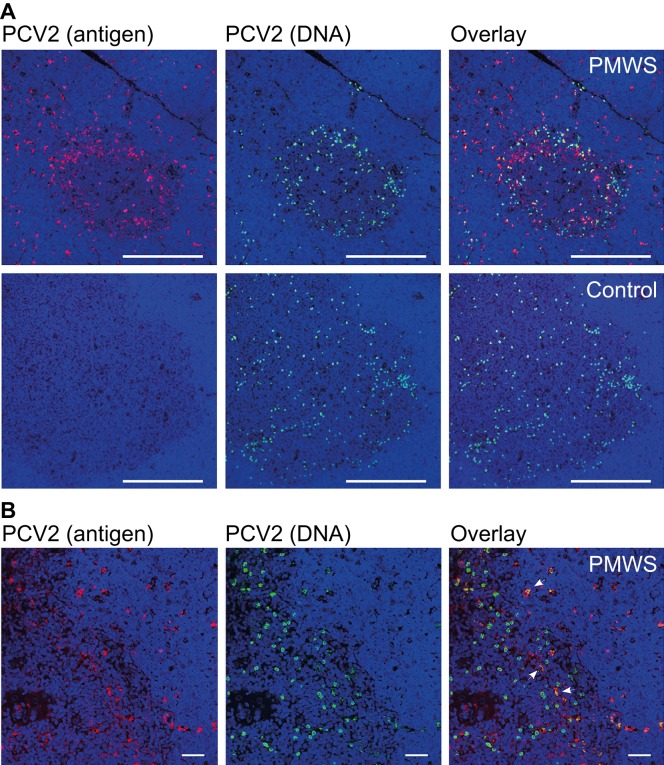
PCV2-ligand carrying APCs and latently PCV2-infected cell populations. (**A**) Comparison between antigen and PCV2 DNA carrying cells located mostly at the medullae. Confocal microscopy comparison of IF and FISH-stained, paraffin-embedded thymus tissue sections from a PMWS and a control pig. Medulla appeared darker with loosely arranged nuclei (blue, DAPI staining) and a 300 μm white bar. (**B**) Higher magnification of (**A**) with cells containing both PCV2 antigen and DNA from a PMWS thymus. White arrows indicate double-stained cells with PCV2 antigen and genomes. Nuclei (DAPI) appear blue, and the white bar is 40 μm. DAPI, 4′,6-diamidino-2-phenylindole.

For a better understanding of the APCs carrying PCV2, we analyzed the presence of PCV2 through a combined approach of IF and FISH using confocal microscopy. This revealed that most PCV2 antigen and DNA did not colocalize in the PMWS thymi (*n*=2) (Figure 3A). A few polygonal-shaped putative single APCs with PCV2 antigen (Figure 1D) contained PCV2 DNA (Figures 3A and 3B). The round-shaped PCV2-DNA^+^ cells were highly infected with PCV2 but without any detectable PCV2 antigen (Figure 3B). This infected PCV2 cell type was also present in other organs of the immune system (data not shown and Ref. 33) and in all other pig thymi, including non-transfected controls (Figure 3A; *n*=32). Additionally, recurrent low viremia in the CysA and control individuals (Figure 1A) without the presence of IgM expression (Table 1) showed the reactivation potential of this internal virus pool. This indicates a seemingly latent PCV2 infection of pigs in the absence of any antigen expression and immune response (Figures 3A and 3B and Table 1). We confirmed this latent PCV2-infected cell prevalence in a larger study of pig fetuses (data not published).

Both cell types appeared in the thymic medulla and concentrated at the corticomedullary junction (Figures 3A and 3B). The higher magnification in Figure 3B confirmed the two cell types distinguishable by shape and signals. Alongside the single, separated APCs, the medullar TEC scaffold also seemed to contribute to PCV2-antigen presentation at the thymic corticomedullary junction (Figure 3B). Interestingly, PCV2-antigen presentation did not end exactly where the medulla borders the thymic cortex, as recognized by the denser nuclear stain in the cortex (Figure 3B). When taken together, the data above indicating latent PCV2-infected cells residing in the medulla and at the corticomedullary junction of the thymus, the new thymic medullary appearance of PCV2 APCs in disease and the specific depletion of SPs pointed to thymocyte negative selection.^2^

### Hypo-responsiveness of Th cells and adaptive tolerance

**Figure 4:**
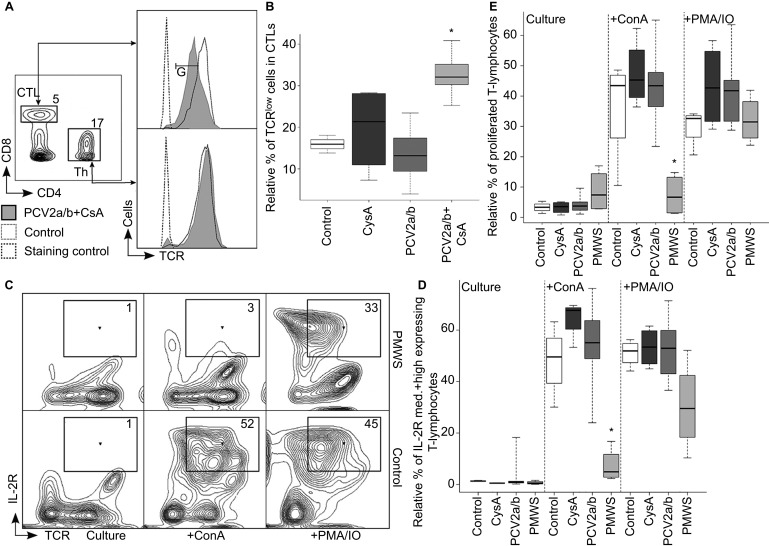
High PCV2 viremia made *in vivo* T-helper cell hypo-responsiveness visible and caused T-cell anergy. (**A**, **B**) The TCR was specifically downregulated on CTLs and not on Ths. (**A**) Three-color flow cytometry with the percentage of total lymphocytes for CTLs and Ths and a TCR^low^ cell gate (gate: G). TCR expression histogram of PCV2a/b+CsA versus control pigs at day 51 p.t. (**B**) TCR^low^ expressing CTLs are significantly more abundant in PCV2a/b+CsA (*n*=5) (PCV2a/b (*n*=9), CysA (*n*=4), control (*n*=3); pigs per group (*n*); *P<0.01, Kruskal–Wallis rank sum test). (**C**–**E**) IL-2R expression and proliferation of T cells in PMWS versus other pig groups. Two-color flow cytometry analysis of *in vitro* cultured and ConA- or PMA/IO-stimulated PBMCs. (**C**, **D**) IL-2R medium (med.) and high expressing T-cell gates (numbers indicate T-cell percentage of medium and high IL-2R expressers). (**D**) Statistical comparison of IL-2R medium- and high-expressing T cells compiled from days 41 and 51 p.t. PMWS T cells did not upregulate IL-2R after receptor-mediated (+ConA) *in vitro* stimuli (PMWS (*n*=4), PCV2a/b (*n*=10), CysA (*n*=4) and control (*n*=4); data points (*n*); (*P<0.002, Kruskal–Wallis rank sum test). (**E**) Statistical comparison of T-cell proliferation compiled from days 41 and 51 p.t. PMWS T cells did not proliferate after receptor-mediated (+ConA) *in vitro* stimuli (PMWS (*n*=4), PCV2a/b (*n*=10), CysA (*n*=4) and control (*n*=4); data points (*n*); (*P<0.005, Kruskal–Wallis rank sum test).

At day 51 p.t., we observed lymphopenia (maximal twofold) in the PMWS pigs that was weak compared to the lymphopenia observed in PCV2-SD field studies.^35^ This may be because the pigs were euthanized early at the onset of diarrhea. Suboptimal antibody responses (Table 1) and persistent PCV2-antigen in the lymphoid organs (Figure 1B and Table 2) led us to inquire whether T-cell defects were involved in disease development. We observed downregulated TCR expression on cytotoxic T cells (CTLs) of the PCV2a/b+CsA pigs (Figure 4A; *n*=5) with the highest viremia (Figure 1A) as a trend as early as day 35 p.t. This phenomenon became significantly pronounced at day 51 p.t. (Figure 4B; *P*<0.01), but was not observed on Ths (Figure 4A). TCR downregulation prevents chronic immune activation of the T cells that otherwise would be detrimental to the host, as observed in human immunodeficiency virus infections.^48^ This indicated to us that the Ths were hypo-responsive to the same antigen levels to which CTLs responded (Figure 4A). This inappropriate Th response explains the observed B-cell defect of PCV2-specific IgM-to-IgG maturation (Table 1) and the inadequate CTL clearance of persistent virus infections (Figure 1B and Table 2). Both B-cell and CTL responses are dependent on a functional interleukin-2 (IL-2)-signaling system central to T-cell help. This can be analyzed in cell culture by activating pig T cells via receptor-mediated signaling, which leads to IL-2-receptor (IL-2R) upregulation and cell proliferation. We observed the failure of IL-2R upregulation on receptor-mediated activated T cells from the PMWS pigs (Figures 4C and 4D: +ConA) as early as day 41 p.t. Notably, T cells from PMWS pigs did not respond differently compared with the controls at day 26 p.t. (data not shown). Bypassing cell surface receptor-mediated signaling *in vitro* rescued the defect in the PMWS pigs' T cells (Figures 4C and 4D: +PMA/IO). Indeed, T cells from PMWS pigs did not proliferate upon *in vitro* receptor-mediated signaling (Figure 4E and Supplementary Figure S2: +ConA), while a bypass of cell surface receptor-mediated signaling induced proliferation (Figure 4E and Supplementary Figure S2: +PMA/IO). These data indicated that the T cells continued to circulate in the blood at least temporarily, although they were non-functional due to *in vivo* anergy, consistent with the adaptive tolerance described in mice under different circumstances.^7^

### PCV2 impacts thymic positive selection with peripheral consequences

**Figure 5:**
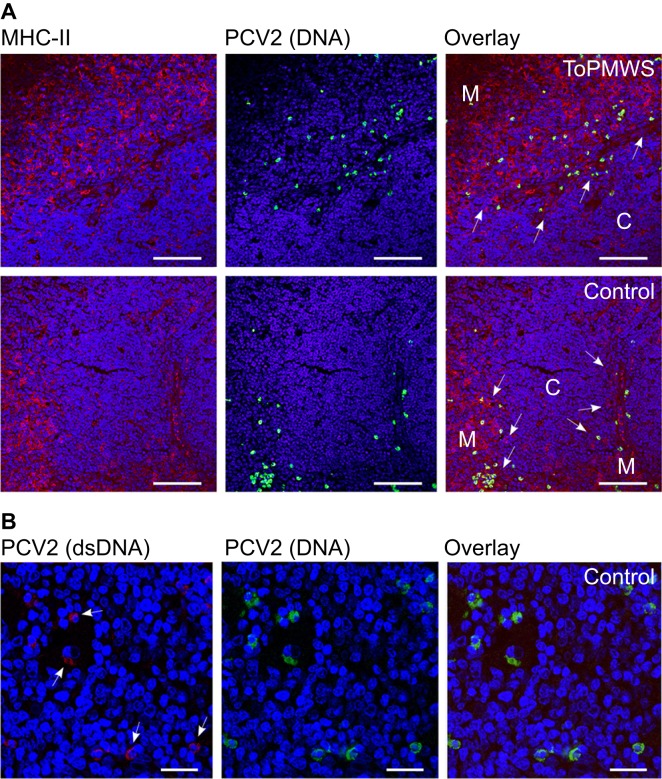
Conventional pig thymi carry generally mixed PCV2-infected cell populations mostly in the medulla and at the corticomedullary junction of the thymus. (**A**) Confocal microscopy comparison of IF and FISH-stained, paraffin-embedded thymus tissue sections. The medulla (white M) compared with the thymus cortex (white C) contains more MHC-II expressing cells. White arrows indicate the corticomedullary junction. Nuclei (DAPI) appear blue with 80 μm white bars. (**B**) Low PCV2 dsDNA signals were present throughout conventional pig thymi. Confocal microscopy of double FISH-stained, paraffin-embedded thymus tissue. Arrows indicate weak PCV2 dsDNA signals. Nuclei (DAPI) appear blue with 20 μm white bars.

Thymi from conventional pigs generally contained cells with latent PCV2 (*n*=32). The cell abundance was roughly the same when comparing PMWS, ToPMWS and control thymi (Figures 3A and 5A). The latently infected cells were mostly round and larger (an average diameter of approximately 10 μm) than the thymocytes, and were located in the medulla and at the corticomedullary junction (Figures 3A and 5A). We found that fetuses commonly carried these latently infected cells early in pig ontogenesis and mostly in the thymus (data not published). Some PCV2-infected cells also carried low concentrations of PCV2 dsDNA (Figure 5B), indicative of possible reactivation and expression of the viral capsid protein that would make PCV2 recognized as ‘self' by the maturing thymocytes.

**Figure 6:**
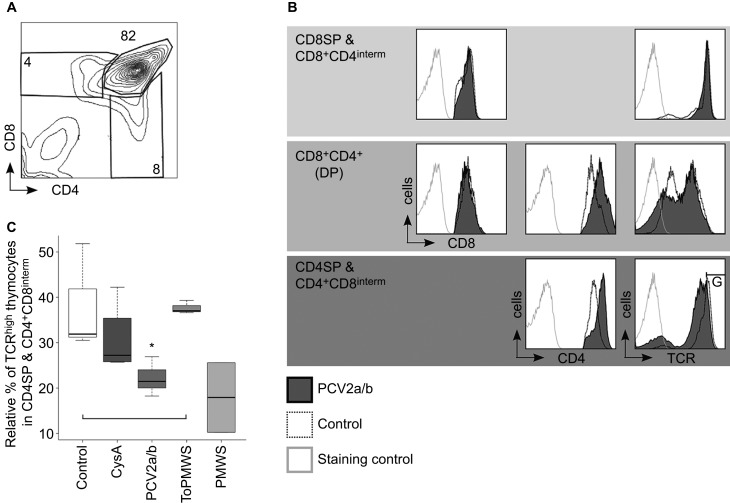
PCV2 reinfection caused reverse CD4 coreceptor-TCR dichotomy in latently infected pigs. (**A**) Contour plot of CD4 versus CD8 coreceptor-expressing control thymocytes with gated cell populations (percentage numbers of total thymocytes). (**B**, **C**) PCV2 productive infections cause thymic reverse CD4 coreceptor-TCR dichotomy on CD4^+^CD8^interm^ (interm: intermediate) cells and CD4SPs. (**B**) Histogram comparisons of CD8 and CD4 coreceptor and TCR expression on PCV2a/b versus control thymocytes by flow cytometry. CD4^+^CD8^interm^ and CD4SPs gated for TCR^high^ expression (gate: G). (**C**) PCV2 reinfection caused TCR downregulation on CD4^+^CD8^interm^ and CD4SPs. Statistical TCR downregulation analysis on PCV2a/b (*n*=9) versus control (*n*=3) or CysA (*n*=4) or ToPMWS (*n*=3); pigs per group (*n*); *P<0.02, Kruskal–Wallis rank sum test; PMWS (*n*=2).

Pig CD4 and CD8 coreceptor-expressing thymocyte populations are similar to mouse and human thymocytes (Figure 6A). The inadequate Th response, as observed in the differential peripheral TCR downregulation of CTLs and not Ths upon exposure to same antigen concentrations in the PCV2a/b+CsA pigs (Figures 4A and 4B), led us to question whether there is evidence for selective maturation defects of CD4 coreceptor expressing precursors in the thymus. With productive infection ≥10^6^ and ≤10^7^ PCV2 per mL blood, we observed modulation of signal module expression at the CD4^+^CD8^interm^ and CD4SPs stage in the thymi of the PCV2a/b pigs (*n*=9). The CD4 coreceptor was additionally upregulated by about 1.5–3 logs of fluorescence intensity from the increase of CD4 coreceptor expression during the DP to SP transition, but the TCR was concomitantly downregulated on CD4SPs and CD4^+^CD8^interm^ cells (Figures 6B and 6C). The TCR and CD8 coreceptor on CD8SPs & CD8^+^CD4^interm^ cells were barely affected (Figure 6B). Considering that upregulation of the CD4 coreceptor is known to promote the selection of low-avidity TCR expressing thymocytes^49^ and CD4-coreceptor lineage committed thymocytes have stringent signaling requirements,^5,50^ our data help to explain the selective maturation defect observed in Th cell hypo-responsiveness.

## DISCUSSION

We present evidence of a new viral host immune evasion strategy by the *Circoviridae* family member PCV2 that involves reprogramming of the T cells in the thymus. Latently PCV2-infected cells and possibly viral capsid expressers are present at the thymic location of thymocyte positive and negative selection. Th cell hypo-responsiveness (Figure 4A) is best explained with viral interference in the special maturation requirements of their thymocyte precursors,^5,50^ which emphasizes the importance of the kinetic signaling model.^4,5^ This explanation is supported by the observation that peripheral viral concentrations specifically influenced CD4-coreceptor/TCR expression at the thymocyte stage of positive selection, and APCs sensed and fed back peripheral viral antigen concentrations into thymocyte development. This was evident in diseased pigs (PMWS) with high viremia (10^9^ viruses/mL blood) and the appearance of new thymic PCV2-ligand-loaded APCs that specifically caused polyclonal deletion of the SPs. We think all of these observations are connected to PCV2 becoming part of the host by adaption, as latently virus-infected cells were found in all adult thymi and even in pig fetuses before the appearance of T-cell development in the thymus (data not published). This explains the initial tolerance of the host immune system towards PCV2, as measured in the control and CysA pigs' recurrent low viremia (Figure 1A) with no PCV2-specific IgMs or IgGs (Table 1). Because of the thymic presence of PCV-infected cells, PCV2 capsid was recognized as self-ligand, and the high-avidity thymocytes that were CD4-coreceptor lineage committed T cells recognizing PCV2 were deleted by clonal negative selection. Remaining low-avidity Ths were not triggered by low peripheral levels of PCV2 in the controls and PCV2a/b pigs. This was observed as an inadequate virus specific affinity maturation of B-cell and CTL responses.

With a second PCV2 infection on top of the endogenous latent PCV2 infection in our experimental design, we noticed that the average viremia with or without immune suppressant cofactors remained mostly constant from day 27 to 52 after initial viral proliferation (Figure 1A). This was surprising in a tolerant host and shows how well the PCV2 propagation cycle has adapted in the pig. Reinfections are a reality due to the high resilience of *Circoviridae* in adverse environments,^51^ and the conducive thymus sanctuary central to virus propagation supports low PCV2 virulence. This viral propagation cycle sustains maximal shedding^31^ and virus dissemination.^31^

With detectable PCV2-antigen in the PMWS thymi, we observed massive polyclonal negative selection of SPs. This massive reduction of SPs went beyond clonal selection and is better defined as polyclonal negative selection. It is conceivable based on mouse studies ^3^ that the new emerging viral capsid APCs are the original peripheral dendritic cells that have acquired PCV2 ‘self-ligand'. With higher viremia (PMWS), these APCs appeared more frequently and were saturated with viral antigen at the corticomedullary junction of the thymus. Taken together and considering the recent evidence in mice of the plasticity of TCR recognition^52,53^ and the sum of many affinity interactions that drive thymocyte selection,^52^ it is not surprising that we observed polyclonal negative selection in the PMWS pigs with an overload of thymic viral capsid presentation that provides many low-affinity interactions that induce apoptosis in maturing TCR^high^ thymocytes. This observed phenomenon does not redefine the thymic negative selection process, but simply reflects dependence on the strength of the signaling-module transmitted signal, as observed *in vitro*.^54^

The polyclonal deletion of SPs interrupted the naïve T-cell supply from the thymus, noted here as modest lymphopenia. The remaining T cells were crippled by chronic TCR engagement,^48^ with high blood concentrations of PCV2 leading to *in vivo* anergy^7^ and the ultimate failure of the adaptive host immune response.

For several decades, possible immune suppression by *Circoviridae* or *Anelloviridae* family members has intrigued scientists. These viruses are highly disseminated throughout healthy young animals and children, and higher viral loads are associated with a variety of disease syndromes. Many of these viruses still have orphan status. Of *Circoviridae*, PCV2 is the simplest member associated with known diseases of these single-stranded DNA viruses. This may make PCV2 pathogenicity mechanisms fundamental to these viruses. We compared PCV2 pathogenicity with the only other well-studied family member, chicken anemia virus (CAV), which is ∼500 nucleotides larger and causes disease in young chickens.^55^ Generalized lymphoid atrophy in PCV2 and CAV is similar, including lymphopenia, suboptimal antibody responses, immunosuppression and thymic atrophy.^56^ Moreover, we found that pig fetuses were latently PCV2-infected in the thymi (data not published), reminiscent of latent CAV thymic tropism.^56,57^ Hardly any PCV2-DNA-containing cells were cortical, which partly contrasted with the cortical PCV2-antigen location in diseased pigs. CAV antigen is also observed in the thymic cortex and at the corticomedullary junction.^57^ Unique to our study is that we combined PCV2-antigen detection by IF with PCV2 genome detection by FISH (Figures 3A, 3B and 5A). This allowed for the differentiation of viral antigen-carrying cells versus latently infected cells. Reduction of CD4- and CD8-expressing thymocytes was also noted in CAV diseased young chickens.^56,57^ This thymocyte depletion was attributed to direct CAV infections that destroyed a precursor thymocyte population;^56^ nevertheless, the authors were analyzing the viral antigen content only in chicken thymi saturated with virus and did not show infected thymocytes by the presence of CAV genomes.

Both *Circoviridae* members modulate CD4 coreceptor expression. Similarly to diseased young chickens,^56^ we found downregulated CD4 coreceptor in two of the PCV2a/b+CsA pigs' thymi (data not shown). More importantly, the CD4 coreceptor was upregulated upon PCV2 transfection (group: PCV2a/b) and the TCR was concomitantly downregulated on the thymocytes. This reverse CD4 coreceptor-TCR dichotomy caused by PCV2 reinfection provided insight about the Th maturation defect with observed Th hypo-responsiveness. This specific hypo-responsiveness makes sense considering that CD4 coreceptor upregulation in transgenic mouse thymus rescued T cells with weakly interacting TCR provoked a skewing of the T-cell populations,^49^ and CD4 coreceptor lineage-committed thymocytes have stringent signaling requirements for differentiation.^5,50^ Similarly, in PCV2a/b pig thymi, higher CD4 coreceptor and lower TCR expression probably elucidated what normally occurs in the background through the thymic presence of mixed PCV2-infected cells (Figure 5B), i.e., the selection of low-avidity TCR-expressing T cells that are CD4-coreceptor lineage committed. This ties in with the observed high levels of antigen presentation in PMWS pig thymi that led to selective TCR downregulation (Figure 6C), although the CD4 coreceptor was unchanged or even downregulated on SPs to escape negative selection.

It is rather disturbing that *Circoviridae* circoviruses, gyroviruses and possibly also cycloviruses, with their highly genomic plasticity and widespread presence among animals and humans, have found a unique niche in the sanctuary of the thymus to modulate the basic requirements of the immune response in even healthy individuals. Our observed skewing of T-helper cell signaling due to CD4 coreceptor/TCR-mediated signal modulation by the virus on maturing thymocytes may also have implications for other infections or even in the formation of tumors and premature aging. In disease, PCV2 infections are often observed together not only with *Anelloviridae* family members but also with other more aggressive pathogenic infections.^31^ Time will tell whether the subtle shifting of maturing thymocytes' basic CD4-coreceptor/TCR signaling requirements, as mediated by the *Circoviridae* family member PCV2, possibly in the context of other *Circoviridae* family member and/or *Anelloviridae* family member infections, is a main contributor to host disease susceptibility in general.

## Figures and Tables

**Table 1:** An overview of infection, treatment and pig parameters

Infection and CsA treatment	IgM	IgM 30d^a^	IgM 51d^a^	IgG	PCV2^b^	PCV2^b^ 30d^a^	PCV2^b^ 51d^a^	Tonsil and PCV2 antigen	Thymus and PCV2 antigen	PMWS
PCV2a/b+CsA										
Females	3/4	2/2	1/2	1/4	3/4	2/2	1/2	c4/4	0/4	0/4
Males	7/7	1/1	3/6	0/7	7/7	1/1	6/6	7/7	2/7	2/7
PCV2a/b										
Females	0/2		0/2	0/2	0/2		0/2	0/2	0/2	0/2
Males	6/6		3/6	0/6	4/6		3/6	c1/6	0/6	0/6
CsA treated pigs^d^	0/7	0/2	0/5	0/7	0/7	0/2	0/5	0/7	0/7	0/7
Untreated pigs^d^	0/6	0/1	0/5	0/6	0/6	0/1	0/5	0/6	0/6	0/6

PCV2-specific antibodies are indicated as IgM or IgG. Numbers are affected pigs compared to total pigs, x/n.

a Day (d) of slaughter and necropsy post-transfection.

b The blood virus content was>10^6^ genomes/mL in at least one of the measurements.

c Two females and one male pig showed only a few anti-PCV2 stained lymphatic cells in proximity to the crypts.

d CsA-treated and untreated pig groups were mixed gender.

**Table 2:** Immunohistochemically determined PCV2-specific antigen content of pig organs

	PCV2a/b+CsA (*n*=5)				
IHC from pig organs	PMWS (*n*=2)	ToPMWS (*n*=3)	PCV2a/b (*n*=9)	CysA (*n*=5)	Control (*n*=5)
Tonsil	+++++	+++/++/++	+**/+^#^	−−−	−−−
Thymus	++++/++	−−−	−−−	−−−	−−−
Lymph nodes	+++	+++/++/+	−−−	−−−	−−−
Ileum (Peyer's patches)	+++	++/+/+	+*	−−−	−−−
Spleen	+++	++/+/+	+*	−−−	−−−
Kidney	−−−	−−−	−−−	−−−	−−−
Lung	++	−−−	−−−	−−−	−−−
Heart	−−−	−−−	−−−	−−−	−−−
Liver	+	−−−	−−−	−−−	−−−
Intestinal (lamina propria)	++/+++	−−−	−−−	−−−	−−−

+++++Very high PCV2-antigen concentrations.

++++high concentrations of PCV2-antigen.

+++moderately high concentrations of PCV2-antigen.

++moderate concentrations of PCV2-antigen.

+low concentrations of PCV2-antigen with only a few lymphatic cells stained.

**2 females, *1 female, ^#^1 male and (*n*) number of pigs.
