# Supplementary material for: T-cell reprogramming through targeted CD4-coreceptor and T-cell receptor expression on maturing thymocytes by latent Circoviridae family member porcine circovirus type 2 cell infections in the thymus
Source: Emerg Microbes Infect. 2015 Mar 11;4(3):e15–. doi: 10.1038/emi.2015.15 (PMC4355439; doi:10.1038/emi.2015.15)
Supplement: Supplementary information [file emi201515x1.pdf]

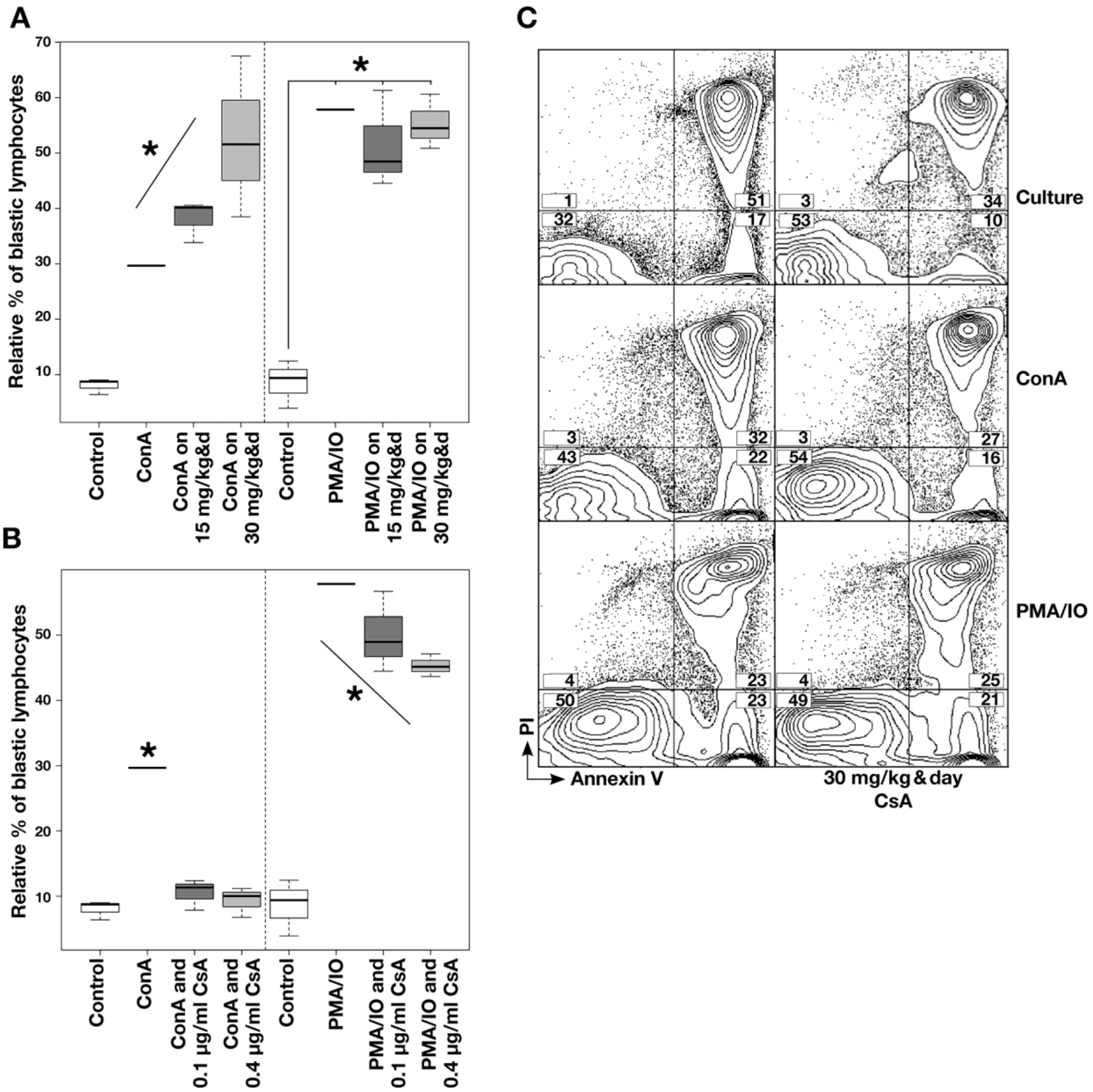

Figure S1 *In vitro* ConA-mediated hyper-stimulation and survival of PBLs from CsA treated pigs.

(**A**, **B**) Size quantification by flow cytometry of *in vitro* stimulated, cultured pig PBLs. Three separate experiments were normalized according to the PBL controls (d for day). (**A**) PBLs of *in vivo* CsA-treated pigs stimulated *in vitro* with ConA or PMA/IO. CsA dose-dependent ConA hyper-stimulation (*P < 0.02, Kruskal-Wallis rank sum test). The PMA/IO combination bypasses receptor-mediated signaling; all stimulated PBLs appeared similarly large and yet significantly different from the unstimulated controls (*P < 0.01, Kruskal-Wallis rank sum test). (**B**) PBLs cultured with different CsA doses and ConA or PMA/IO stimulation. Both ConA and PMA/IO stimulation are inhibited by *in vitro* addition of CsA (*P < 0.01, Kruskal-Wallis rank sum test). PMA/IO stimulation is inhibited in a CsA dose-dependent fashion (*P < 0.02; Kruskal-Wallis rank sum test).

(**C**) *In vitro* cultured or stimulated PBLs from 30 mg/kg/day-treated pigs have similar parameters to PBLs from control pigs. Apoptosis was measured with PI versus

annexin V by flow cytometry. Numbers indicate the sector percentage of total lymphocytes.

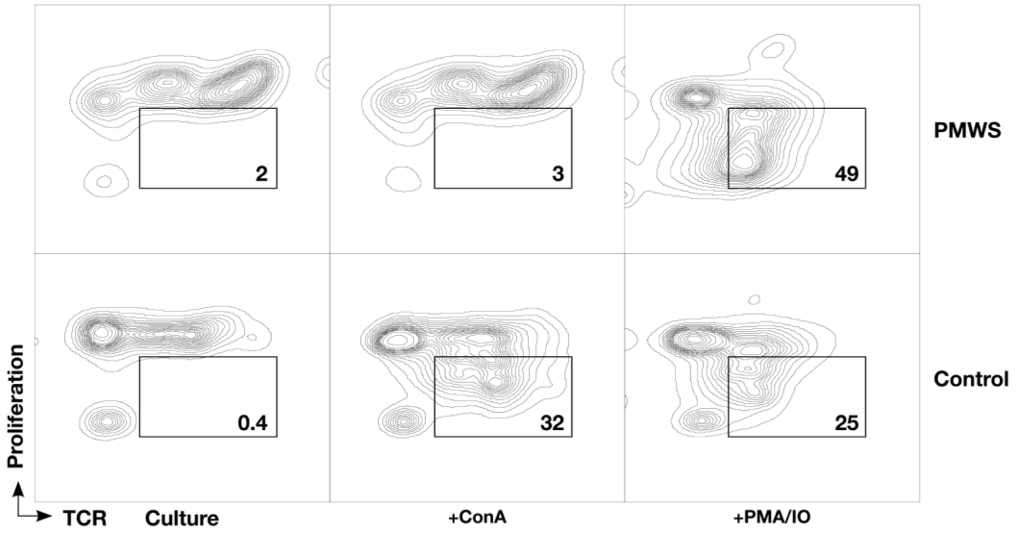

Figure S2 T-cells from PMWS pigs did not proliferate *in vitro* upon receptor-mediated signaling.

PMWS versus control pig T-cell proliferation. Two-color flow cytometric analysis of *in vitro* cultured and ConA- or PMA/IO-stimulated PBMCs. Proliferation of TCR$^+$ cells are gated (numbers indicate the percentage of proliferating T-cells).
